# S82. Proffered paper: In-vivo testing of PSMA-targeted T-cell immunotherapy for prostate cancer

**DOI:** 10.1186/2051-1426-2-S2-I20

**Published:** 2014-03-12

**Authors:** Z Liu, SB Papa, J Morris, J Maher

**Affiliations:** 1 Kings College London and Guys and St Thomas' NHS Foundation Trust, Cancer Studies, London, UK

## Introduction

Bone is the most common site for metastasis in human prostate cancer patients. Skeletal metastases are a significant cause of morbidity and mortality and overall greatly affect the quality of life of prostate cancer patients. Despite advances in our understanding of the biology of primary prostate tumours, our knowledge of how and why secondary tumours derived from prostate cancer cells preferentially localise in bone remains limited. Examining the impact of these facets of bone metastasis in vivo remains a significant challenge, as animal models that closely mimic the natural history and malignant progression of clinical prostate cancer are not available.

## Objectives

To develop an animal model of human metastatic prostate cancer. Once a model has been developed and optimised, it was this to test efficacy of immunotherapy using T-cells that have been genetically targeted against prostate-specific membrane antigen (PSMA).

## Material and methods

Using PCR, western blot, flow cytometry and ELISA, we performed functional anaylsis of fucosyltransferase 3(FT3) in PC3LN3(PL)and PC3LN3-PSMA (PLP) tumour cell lines. In *vivo* bioluminescent imaging (BLI) was used to detect metastases.

## Results

In preliminary studies, we have observed that delivery of a FT3-encoding retroviral vector to PL and PLP enables them to express sialyl Lewis X and to acquire E-selectin binding activity. We also showed that FT3 promotes increased PLP motility and invasiveness *in vitro*. Bioluminescent animal model of metastasised prostate cancer is established to determine the effect of this upon their pattern of metastatic spread in SCID Beige mice.

## Conclusion

We have established an *in-vivo* model of PSMA-expressing prostate cancer. This will serve as a platform to test immunotherapy using P28z+ T-cells.

